# Exploring bispectral index as an alternative to polysomnography for long-term sleep monitoring in disorders of consciousness

**DOI:** 10.3389/fneur.2024.1408890

**Published:** 2024-10-14

**Authors:** Qianqian Ge, Kaitian Chen, Qinghua Li, Yutong Zhuang, Peijian Sun, Long Xu, Jianghong He

**Affiliations:** ^1^Department of Neurosurgery, Beijing Tiantan Hospital, Capital Medical University, Beijing, China; ^2^Nanfang Hospital, The First School of Clinical Medicine, Southern Medical University, Guangzhou, China; ^3^Department of Anesthesiology, Beijing Ditan Hospital, Capital Medical University, Beijing, China

**Keywords:** disorders of consciousness, bispectral index, electroencephalographic, sleep monitoring, sleep, minimally conscious state (MCS), unresponsive wakefulness syndrome (UWS)

## Abstract

**Background:**

Sleep plays a crucial role in neuroplasticity and recovery from brain injury, particularly in disorders of consciousness (DoC), including unresponsive wakefulness syndrome (UWS) and minimally conscious state (MCS). Traditional sleep monitoring methods like polysomnography (PSG) are complex and often impractical for long-term use in clinical settings.

**Target:**

This study aimed to explore the utility of the Bispectral Index (BIS) as a more practical alternative for monitoring sleep patterns in DoC patients.

**Methods:**

We conducted simultaneous PSG and BIS monitoring on 38 DoC patients (19 UWS and 19 MCS). The study focused on analyzing sleep timing distribution, the effectiveness of BIS in differentiating sleep stages, and its correlation with consciousness levels.

**Results:**

Our findings revealed that DoC patients exhibited irregular and fragmented sleep patterns, necessitating extended monitoring periods. The BIS effectively differentiated various sleep stages, with significant differences in BIS values observed across these stages. However, BIS values did not show significant differences between UWS and MCS patients, indicating that BIS primarily indicates wakefulness rather than cognitive awareness. DoC patients have disturbed sleep-wake cycles that require prolonged monitoring. BIS can well distinguish sleep stages in DoC patients, and the distribution of values is similar to that of normal subjects. However, BIS could not distinguish the level of consciousness of DoC patients.

**Conclusion:**

The study demonstrates the potential of BIS as a practical tool for long-term sleep monitoring in DoC patients, offering a less intrusive alternative to traditional methods.

## 1 Introduction

Sleep is integral to neuroplasticity and recovery from brain injury, particularly in disorders of consciousness (DoC), including unresponsive wakefulness syndrome/vegetative state (UWS/VS) and minimally conscious state (MCS). Studies have consistently shown that sleep patterns in DoC patients are irregular and fragmented, with significant variations in sleep–wake cycles and sleep structure (1). Studies have found that these abnormalities are more pronounced in patients with a lower level of consciousness (2), such as UWS patients (3). The presence of these sleep abnormalities holds diagnostic value for assessing the level of consciousness in DOC patients. Sleep patterns not only have diagnostic value for assessing the level of consciousness at the time of assessment, but they also hold significant value for prognosis and treatment in cases of prolonged DoC (4). The presence of organized sleep–wake patterns and more sleep spindles has been linked to better clinical outcomes (5). The latest European guidelines also recommend the use of sleep EEG for differentiation between VS/UWS and MCS as part of multimodal assessment (6). This underscores the need for effective sleep monitoring in DoC patients.

These abnormalities in sleep architecture are often monitored by the complexity of polysomnography (PSG) (1), the standard method for sleep assessment. However, PSG is complex and often conflicts with clinical care due to its cumbersome nature for long-term monitoring (4). However, patients’ sleep cycles are not primarily nocturnal (3), and monitoring only during the night would provide incomplete results. The presence of alternating periods of eyes-open/eyes-closed cycles does not necessarily imply the preservation of electrophysiological sleep architecture in DoC patients (7). Therefore, it is also challenging to determine the timing of sleep monitoring based solely on observing eye opening and closing. Especially when attempting to assess treatment effectiveness based on changes in sleep patterns, longer periods (even more than 24 h) of continuous monitoring are needed. Therefore, it is necessary to explore a simpler and more convenient method of sleep monitoring that does not interfere with daily rehabilitation activities.

The bispectral index (BIS) is a multifactorial EEG-based measurement that provides a dimensionless score between 0 and 100, correlating with levels of consciousness, which is calculated through a proprietary algorithm combining various EEG features (8). BIS commonly used for monitoring the depth of anesthesia (9), has shown potential in reflecting sleep depth (10), suggesting its utility in less intrusive, long-term sleep monitoring method. There have been studies attempting to use the BIS for sleep monitoring in the intensive care unit (ICU) to assess the impact of sleep on postoperative delirium (11), suggesting that using BIS is a feasible tool for monitoring sleep quality in mechanically ventilated patients in the ICU. This presents an opportunity to explore a more practical approach to sleep assessment for DoC patients, which could aid in their diagnosis and prognosis without the complexities associated with PSG. However, to the best of our knowledge, there is no existing research specifically addressing the BIS monitoring values and their relationship with sleep in patients with DoC.

DoC patients require less invasive and more convenient long-term sleep monitoring, and BIS shows potential as an easy-to-use real-time index for this purpose. However, due to significant differences in brain activity between DoC patients and healthy individuals, it remains uncertain whether BIS can be effective in this specific population. Our study aims to conduct simultaneous PSG and BIS monitoring in DoC patients. This dual approach will allow us to assess the feasibility of using BIS as a simpler alternative for long-term sleep monitoring, and to explore the relationship between BIS readings and consciousness levels.

## 2 Methods

### 2.1 Patients

This study involved 38 DoC patients admitted to Beijing Tiantan Hospital between December 2021 and August 2022. Patients were included according to the following criteria: (1) diagnosis as DoC; (2) age > 14 years regardless of sex; (3) duration of disease more than 1 month; (4) caused by acute conditions such as trauma, cerebral hemorrhage, or ischemic hypoxia. Exclusion criteria were as follows: (1) concurrent neurodegenerative diseases and life-threatening diseases; (2) expected survival time less than 3 months; (3) severe cardiac, pulmonary, hepatic, or renal dysfunction; (4) epilepsy that cannot be controlled with medication. The DoC patients were scored using the revised Coma Recovery Scale (CRS-R) by trained and experienced neurologists. One week before the experiment, each patient received at least 3 times of CRS-R assessment, and the highest score was taken for the diagnosis. The study was conducted in accordance with the ethical standards of the Declaration of Helsinki and was approved by ethics committee of Beijing Tiantan Hospital in November 2021 (No. KYSQ 2021-396-01). The patients and their legal representatives were informed about the study content before study enrollment and gave their written consent.

### 2.2 Polysomnography recordings and sleep stage classification

Patients underwent continuous PSG recording including 32 channels of EEG; two channels of electrooculography (EOG), one channel of electromyography (EMG), and video recording. Vital signs during sleep recording were monitored using electrocardiography (ECG). The data were recorded at a sampling rate of 512 Hz. A total of more than 5 h of sleep time was recorded. All recordings were made in the wards which maintain normal medical conditions. The data were recorded at a sampling rate of 500 Hz using a polysomnographic device (Nicolet EEG V32, Natus Neurology, USA) with 32 Ag/AgCl electrodes based on the international standard 10–20 system setup. All electrodes were set with FCz as the reference electrode and AFz as the ground electrode. The impedance between the electrodes and the patient’s skin was always kept below 10 kΩ.

Two independent raters scored the data under the supervision of the most experienced third person. All raters had previous experience in the classification of sleep stages and were familiar with the essential characteristics of patient groups. Importantly, the raters were blinded to the UWS/MCS status of the patients to prevent any potential bias in scoring. The sleep stages were visually scored for 30-s epochs according to the new guidelines of the American Academy of Sleep Medicine (12). Given the specific challenges in DoC patients, adjustments were made to the classification criteria. We differentiated between the patient’s inherent slow waves and sleep-related delta waves by using 32-channel EEG. The amplitude criterion for slow waves (> 75 μV) was not strictly followed due to the influence of extracerebral factors on EEG amplitude (2). Furthermore, the spindle frequency was expanded to 6–16 Hz, as research suggests that DoC patients may exhibit slower spindles (7). The sleep architecture consists of stage N1, stage N2, stage N3, and REM sleep.

### 2.3 Bispectral index monitoring

BIS data were collected with a BIS Quatro (Medtronic, Ireland) attached with BIS Vista Monitor version 3.01. The adhesive electrodes were applied to the unilateral forehead according to standard placement guidelines, with sensor 1 positioned in the center of the forehead, about 5 cm above the nose girder, sensor 4 just above the eyebrow, and sensor 3 on the temple, between the corner of the eye and the hairline. The data recorded included several parameters, such as electrode impedances, signal quality index (SQI), BIS values, and EMG measured in decibels (dB). During the analysis, segments of BIS data lasting 5 s with SQI values less than 50% were excluded. Overall, 10.2% of the BIS data were excluded from the analysis due to low SQI values. BIS data were recorded in real-time mode, with information extracted every 1 min. To ensure accurate data alignment during later analysis, the time and date settings of the BIS Vista Monitor were synchronized with the time of PSG.

### 2.4 Data analysis

Quantitative data were assessed for normality using the Shapiro–Wilk test and reported as mean ± standard deviation (SD) or median (interquartile range, IQR) when appropriate. Qualitative data were presented as counts and percentages. Due to the presence of multiple epochs for each sleep stage in each patient, we used the average value of each patient for statistical comparisons. The significance level was set at a *p*-value of less than 0.05. Independent sample *t*-test was used to detect differences in BIS values under different sleep stages. The receiver operating characteristic (ROC) were applied to assess the predictive ability of the BIS index for sleep monitoring. Sensitivity, specificity, and the cutoff value of the BIS index were estimated using the Youden Index. For all statistical analyses, SPSS (version 26, IBM) was utilized. The visualization of the images was created using the tools available on the Hiplot.^1^

## 3 Results

### 3.1 Patients information

Among 38 DOC patients (19 MCS and 19 UWS), 23 patients were male and 15 patients were female (Table 1). Their age was 45.0 ± 16.6 years, and their CRS-R score was 8.0 ± 2.6. Etiological factors included cerebral hemorrhage(*n* = 17), craniocerebral trauma (*n* = 12), anoxic encephalopathy (*n* = 7), and other (*n* = 2). The median disease duration was 5 months (interquartile range, 2–9 months).

**Table 1:** Clinical characteristics between the UWS and MCS patients.

	UWS	MCS	*p*-value
	*n* = 22	*n* = 16	
Age, year	50.0 [29.5–58.5]	41.5 [33.3–56.0]	0.63
Gender			0.74
Male	14 (63.6%)	9 (56.2%)	
Female	8 (36.4%)	7 (43.8%)	
Duration, months	4.8 [2.2–8.7]	5.0 [2.4–8.3]	1.00
Etiology			0.15
TBI	8 (36.4%)	4 (25.0%)	
ICH	7 (31.8%)	10 (62.5)	
Other	7 (31.8%)	2 (12.5%)	
CRS-R	7 [5–7]	9 [8–10]	<0.00

UWS, unresponsive wakefulness syndrome; MCS, minimally conscious state; TBI, traumatic brain injury; ICH, intracerebral hemorrhage; CRS-R, revised JFK coma recovery scale. Data are given as median [IQR] for continuous variables and as count (percentages) for categorical variables. Wilcoxon–MannWhitney test was used to compare groups for continuous variables. Chi-square test was used to compare groups for categorical variables.

### 3.2 Analysis of sleep timing distribution and influencing factors in patients with DoC

**Figure 1:**
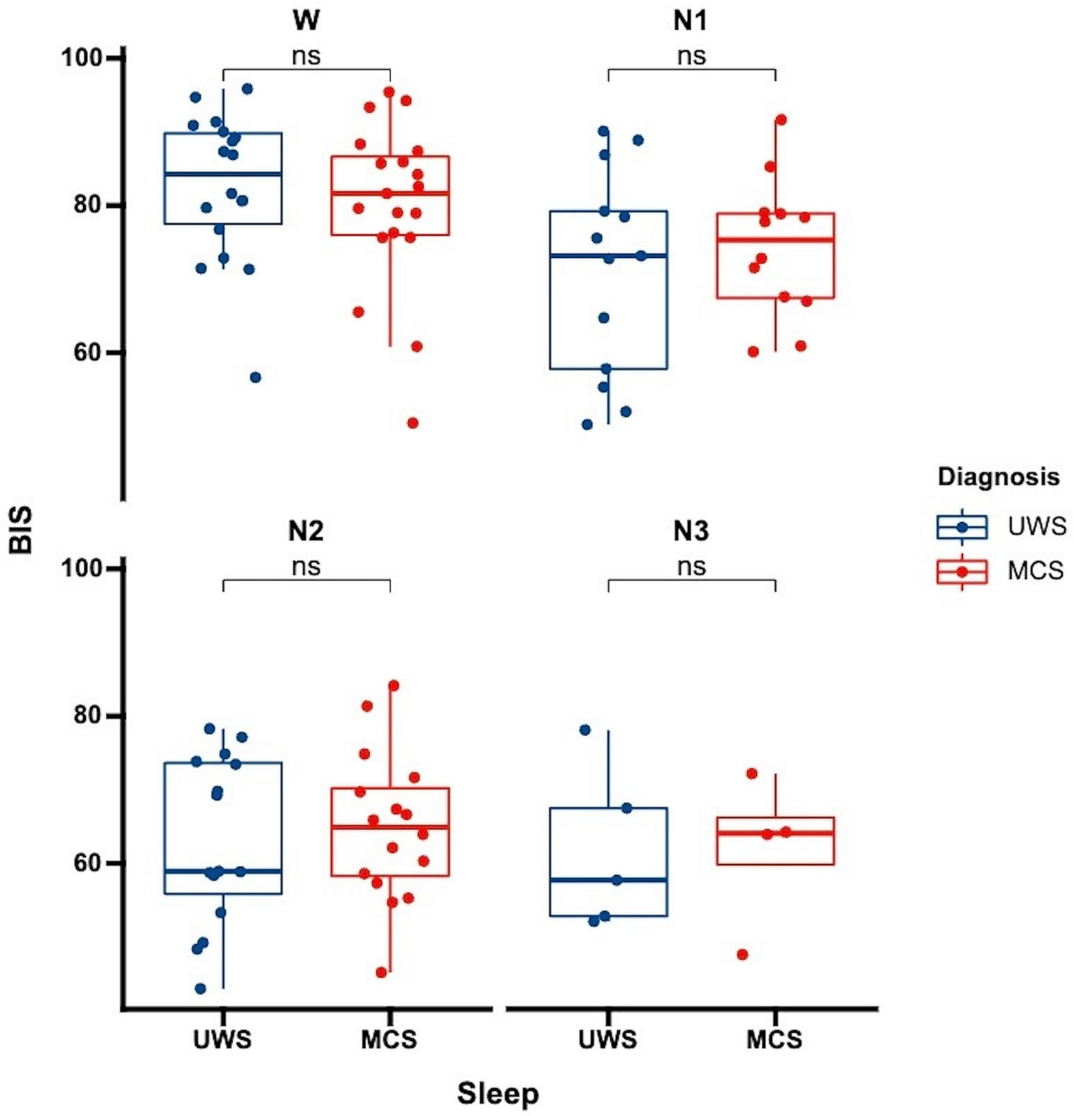
Differences in BIS distribution across sleep stages among patients with varying levels of consciousness each data point represents the average BIS value for an individual patient in a specific sleep stage. UWS, unresponsive wakefulness syndrome; MCS, minimally conscious state; W, wake; N1, non-rapid eye movement stage 1; N2, non-rapid eye movement stage 2; N3, non-rapid eye movement stage 3; ns: no significance.

To identify the optimal time frame for sleep monitoring, we charted the proportion of patients in different sleep stages at different time points through the night (Figure 1). It was observed that patients with DoC did not have a concentrated sleep onset time under routine care conditions. They did not predominantly fall asleep around environmental light changes (around 8 PM sunset, or around 10 PM when the ward lights were turned off). Even though we started the record at 5 PM, 17% of the patients were already asleep, making it challenging to accurately record their sleep onset time. The proportion of patients falling asleep gradually increased from 5:30 PM to 1:30 AM, yet only a maximum of 80% were asleep by around 1:40 AM. Around 0:45 AM, sleep was commonly disrupted due to the nurses’ nighttime rounds. From 2 to 4 AM, most patients experienced sleep interruptions due to nighttime care activities like repositioning, with an absence of deep sleep. Post 4 AM, as the nursing activities ceased, a significant number of patients (50%) re-entered deep sleep. However, with the onset of the nurses’ morning activities around 5 AM, the proportion of sleeping patients once again decreased.

### 3.3 Evaluating BIS as indicators of sleep depth in DoC patients

**Figure 2:**
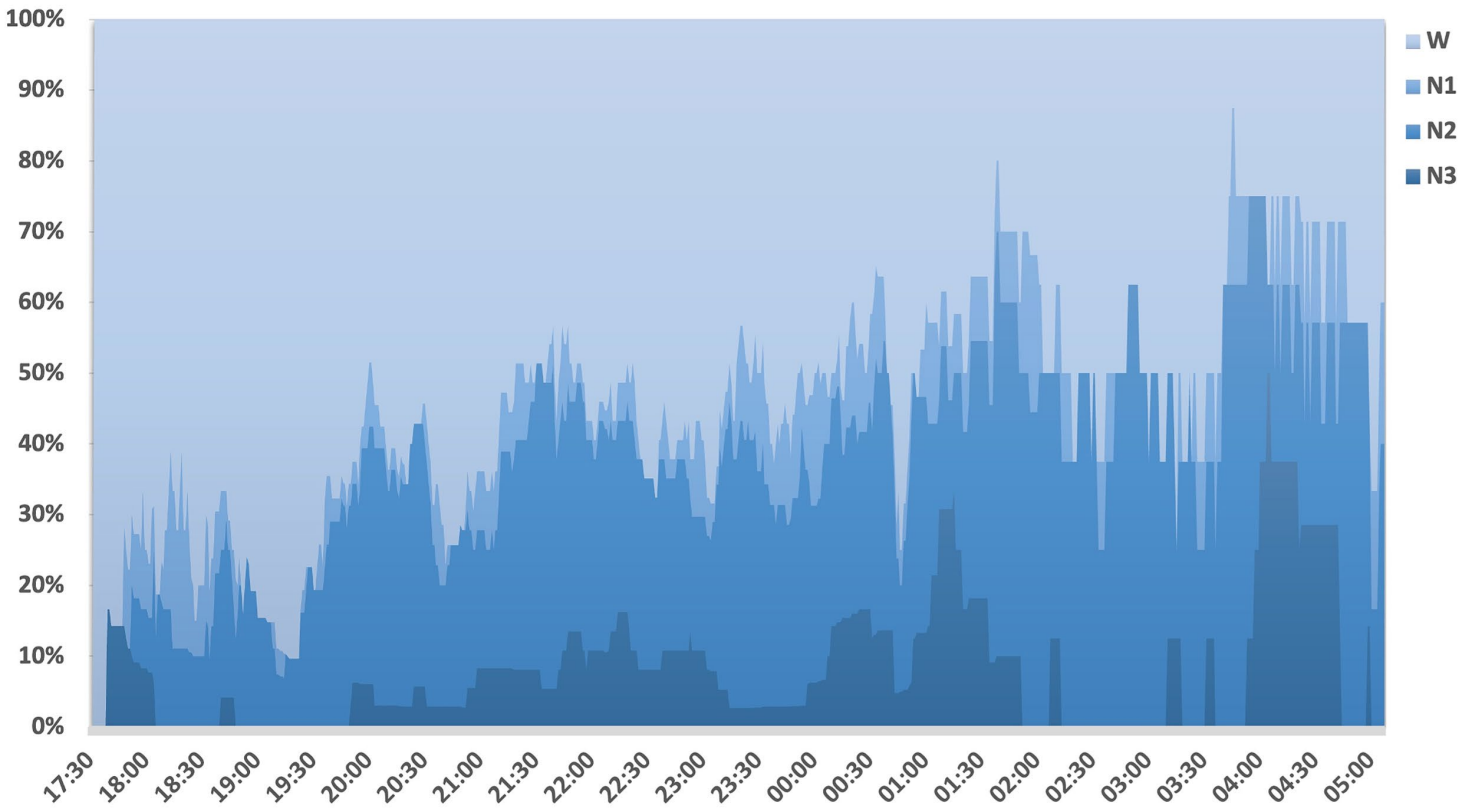
Sleep distribution pattern in patients with disorders of consciousness the sleep states of DoC patients are displayed in percentage form, recorded over different periods, with colors indicating various sleep stages. It is observed that the number of patients falling asleep gradually increases from 5 PM to 4 AM, and the number entering deep sleep also increases, but this increase is gradual and not concentrated in any specific period. Sleep is commonly disrupted between 1 and 3 AM due to nocturnal nursing activities. At around 5 AM, there is a concentrated onset of awakening among the patients.

To determine whether the BIS reflects the depth of sleep in patients, we compared the average BIS values across different sleep stages for the patients. The results revealed significant differences in BIS values between various stages of sleep, except between N2 and N3 stages (*p* = 0.58). Notably, there were significant differences between wakefulness and N1 (*p* < 0.01), wakefulness and N2 (*p* < 0.01), wakefulness and N3 (*p* < 0.01), N1 and N2 (*p* < 0.01), and N1 and N3 (*p* = 0.02), suggesting that BIS values have the potential to reflect sleep depth (Figure 2A). The area under the curve (AUC) for predicting sleep using BIS values was 0.791 (0.784–0.798, *p* < 0.01). A BIS value of 80 as a threshold for predicting sleep and wakefulness yielded a sensitivity of 65.4%, a specificity of 81.3%, a positive predictive value (PPV) of 83.1%, and a negative predictive value (NPV) of 62.6% (Figure 2B).

### 3.4 Comparison of BIS values across different levels of consciousness in DoC patients

**Figure 3:**
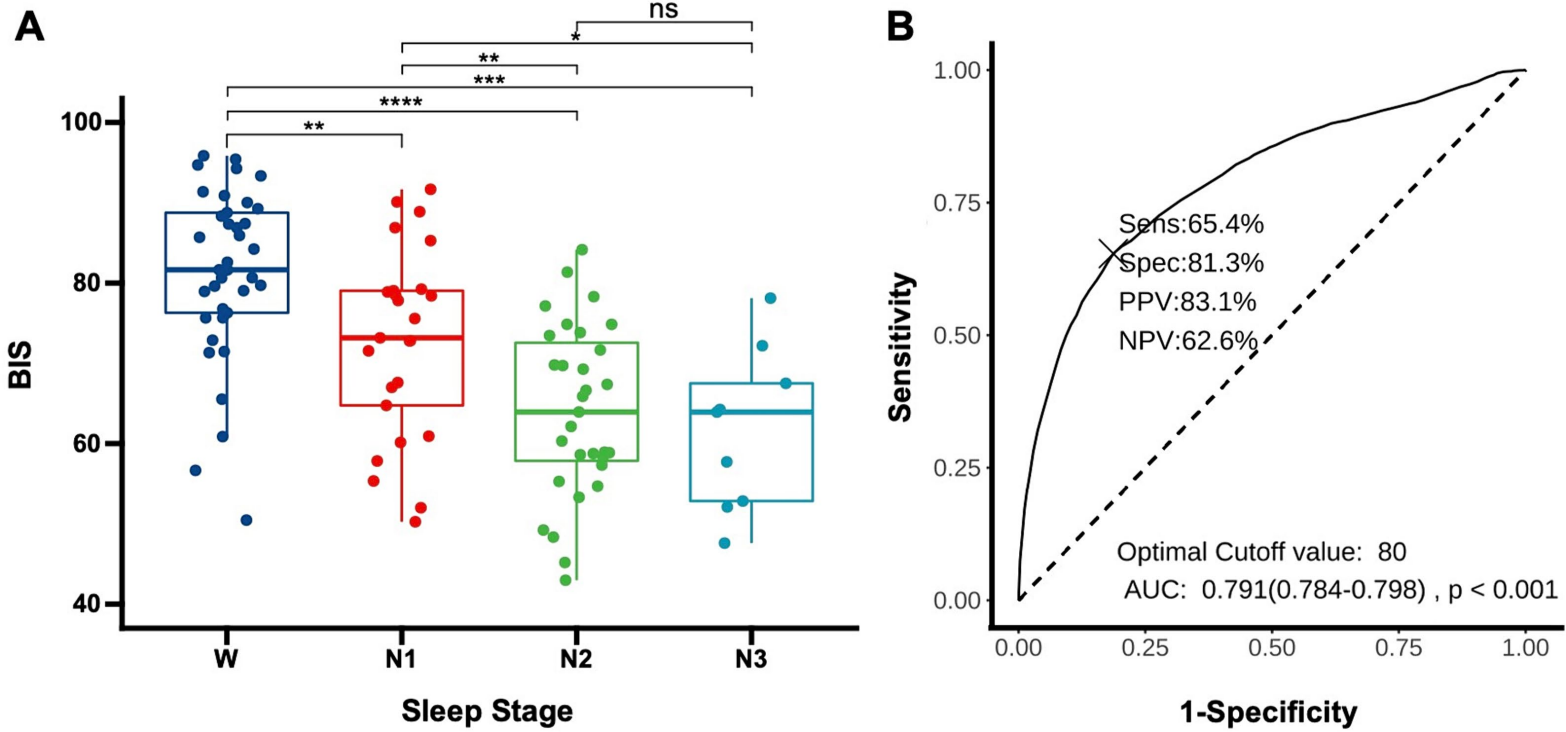
Using BIS to determine sleep depth **(A)** distribution of BIS values in DoC patients across different sleep stages. Each data point represents the average BIS value for an individual patient in a specific sleep stage (W: wake, N1: non-rapid eye movement stage 1, N2: non-rapid eye movement stage 2, N3: non-rapid eye movement stage 3). *p < 0.05, **p < 0.01, ***p < 0.001, ****p < 0.0001; **(B)** Receiver operating characteristic for predicting sleep onset using BIS, AUC, area under the curve; Sens, sensitivity; Spec, specificity; PPV, positive predictive value; NPV, negative predictive value.

We further analyzed whether there were differences in BIS values among patients with varying levels of consciousness. Our analysis showed no significant differences in BIS values between UWS and MCS patients. This was consistent both for the overall average BIS values without distinguishing sleep stages (*p* = 0.81) and when considering specific sleep stages (Figure 3, wakefulness *p* = 0.47, N1 *p* = 0.52, N2 *p* = 0.63, N3 *p* = 0.96). This finding suggests that while BIS is effective in differentiating sleep stages, it may not be sensitive to distinguish between different levels of consciousness within the DoC spectrum.

## 4 Discussion

In our study, we uncovered three key findings regarding sleep monitoring in patients with DoC. Firstly, these patients exhibit unique sleep patterns, characterized by delayed sleep onset and frequent disruptions, necessitating extended monitoring, possibly beyond 24 h, to fully capture their sleep dynamics. Secondly, the BIS emerged as a promising tool for assessing sleep depth, effectively differentiating between various sleep stages, which highlights its potential as a convenient and less intrusive alternative to traditional sleep monitoring methods. However, our third finding indicates that while BIS is effective in reflecting sleep depth, it does not correlate with the patients’ levels of consciousness.

Extensive research has highlighted a significant correlation between the sleep cycles of patients with DoC and their levels of consciousness (13). Notably, healthy individuals and MCS patients typically exhibit more nocturnal sleep than daytime sleep, a pattern less evident in UWS patients (2). This distinct sleep cycle in DoC patients, coupled with varied patterns of brain activity (ranging from non-cyclic to multi-cyclic), is intricately linked to their consciousness levels (7, 2, 14). Accurately gauging sleep duration and cycle patterns necessitates extended monitoring, ideally over 24 h. The presence, absence, or characteristics of specific sleep stages (6, 15) and specific waveforms (in terms of amplitude or rhythm) (3, 16) are also indicative of consciousness levels. For example, higher density of spindle wave occurrence and distinctive frequency and amplitude characteristics of spindles may be indicative of improved consciousness status and a more favorable prognosis for patients (17). The presence of slow-wave sleep (SWS) or REM sleep is of significant value in distinguishing between VS/UWS and MCS (6). The ability to accurately identify these sleep stages and waveforms hinges on capturing the relevant sleep periods within the recording timeframe. There are also studies indicating that continuous EEG (30–48 h) provides more information compared to standard EEG (18).

Our study demonstrates that, at a group level, DoC patients still retain a certain circadian rhythm, with an increasing number of patients sleeping at night. However, the onset of sleep among these patients is notably dispersed, lacking a concentrated interval and showing minimal correlation with environmental factors like lighting. This dispersion poses a challenge in pinpointing the optimal start time for effective sleep monitoring in DoC patients. Moreover, our findings reveal that routine therapeutic interventions and nursing activities disrupt the sleep cycles of DoC patients, with interruptions in sleep cycles and deep sleep phases. Previous literature corroborates that under typical clinical conditions, DoC patients’ sleep cycles are disrupted, deviating from the standard 24-h cycle (19). Consequently, even 24-h recordings may not adequately capture the full extent of patients’ sleep cycles. The challenges of long-term PSG in clinical settings are noteworthy, particularly its impact on routine patient care and rehabilitation activities. To maintain data integrity, it often becomes necessary to reduce or temporarily halt various nursing and rehabilitation tasks, which can be detrimental to patient recovery. This difficulty in recording, combined with the disruption to standard diagnostic and therapeutic activities, complicates the feasibility of more extended or frequent monitoring to evaluate patient responses to treatments. This situation underscores the urgent need for more streamlined, non-intrusive sleep monitoring methods in clinical practice, catering to the unique needs of DoC patients.

Our study demonstrated that BIS values significantly varied across different sleep stages in DoC patients. This finding indicates BIS’s effectiveness as a promising and less intrusive tool for monitoring sleep depth. The overall reduction in cortical activity during sleep naturally leads to the BIS being an effective indicator of sleep depth, which has been corroborated by numerous studies in patients with normal consciousness (20, 21). Giménez et.al has shown that BIS values exhibit significant differences across various sleep stages, and certain metrics can demonstrate the differences before and after sleep deprivation. After sleep deprivation, the average BIS values during the SWS phase are significantly lower than those during the baseline night (10). The study by Chirakalwasan and colleagues demonstrates that BIS monitoring is a feasible tool for assessing sleep quality in postoperative ICU patients, and identified an optimal discrimination threshold of 75 for BIS, with a sensitivity of 68% and a specificity of 56% (11). Our results indicate that the optimal discrimination threshold for the BIS is 80, and this threshold demonstrated higher specificity (81.3%) in our study. This finding aligns with previous results obtained from studies involving normal sleep volunteers, sleep apnea/hypopnea syndrome patients, sleep-deprived anesthesiologists, ICU patients, and patients undergoing elective thoracic surgery (22–26).

BIS monitoring’s convenience and its ability to display real-time values enable even non-EEG specialists to swiftly assess a patient’s condition. This is particularly advantageous for DoC patients, who often exhibit significant fluctuations in consciousness (27), which might result from their unstable sleep rhythms. While it’s feasible to assess consciousness levels during sleep through awakening methods (28), such assessments are prone to inaccuracies, leading to a high misdiagnosis rate in behavioral evaluations (29). Additionally, conducting rehabilitation treatments during sleep can be less effective (30) and may interfere with the patient’s much-needed rest. BIS monitoring allows for the immediate and accurate determination of the patient’s current state, facilitating the selection of optimal times for both assessment and treatment. Furthermore, the unobtrusive and non-disruptive nature of BIS monitoring enables longer monitoring durations, crucial for tracking changes associated with various treatments. Research conducted by Anglade et al. demonstrates that in DoC patients, sleep cycles under dynamic daylight conditions tend to align more closely with the normal 24-h rhythm, exhibiting enhanced clarity and stability (19). Notably, interventions like transcranial direct current stimulation (tDCS) have been shown to improve sleep structure. They significantly extend the total sleep time in VS patients with sleep cycles and contribute to improved CRS-R scores (4). This evidence suggests that some therapeutic approaches can lead to modifications in sleep cycles, meanwhile, the presence or absence of these cycles is closely related to treatment outcomes. Consequently, the integration of long-term sleep structure monitoring during treatment is of paramount importance, offering valuable insights into the efficacy of therapeutic interventions. Furthermore, based on our findings, we suggest that long-term BIS monitoring can be effectively used in DoC patients. Assessments and treatments could be more optimally timed when BIS values exceed 80, potentially enhancing the accuracy and effectiveness of interventions.

DoC Patients exhibit significant differences in brain function compared to healthy individuals. In these patients, there is a decrease in alpha waves and an increase in delta wave power (31), with reduced complexity in electroencephalographic patterns (32). Additionally, there is a diminished connectivity in brain function across multiple regions (33). Some studies have suggested a correlation between BIS scores and neurological status in non-sedated ICU patients, indicating that higher BIS scores might be associated with better neurological function (34). Conversely, lower BIS values have been observed in ICU patients with neurological disorders, such as stroke or traumatic brain injury, which could lead to inaccurate sleep measurements (21). A study of trance hypnosis also demonstrated a correlation between the depth of hypnosis and a decrease in BIS and cerebral state index. This suggests that BIS may be responsive to changes in consciousness (35). However, our study presents a different perspective, showing that patients with varying levels of consciousness demonstrate similar BIS values across different sleep stages. The range of BIS values for DoC patients across wakefulness and sleep stages are similar to those seen in prior studies in ICU patients without central nervous system disorders (11, 22). Our results indicate that the optimal BIS value for distinguishing between sleep and wakefulness in both UWS and MCS patients is around 80, consistent with the threshold previously established in studies involving non-medicated healthy volunteers (26). Previous research has indicated a significant correlation between BIS values and neurological scores in acutely ill critical patients (34). This stands in contrast to our findings, which may be attributed to our patient cohort being in a chronic stage. The decline in neurological function during the acute phase and the loss of consciousness or cognitive decline in the chronic phase are not share the same mechanisms. Moreover, it’s possible that the BIS may only indicate wakefulness but not cognitive function. Consequently, the interpretation of “consciousness” in anesthesia and sleep studies may not be directly applicable to the context of prolonged DoC. Furthermore, this highlights the limitations of BIS in predicting patient outcomes. While PSG provides detailed information such as specific spindle wave rhythms (7), slow-wave amplitude (6), and wakefulness alpha power (4)—all of which are valuable for assessing prognosis—BIS reduces this complexity to a single value. Although BIS is a practical tool for preliminary monitoring of sleep–wake cycles and can guide the timing of assessments and therapeutic interventions, it does not provide sufficient detail for diagnostic and prognostic purposes.

Our study has several limitations that should be acknowledged. The duration of sleep monitoring in our study was relatively short, which may not accurately reflect the full daily sleep–wake rhythm of DoC patients. Due to the partial absence of sleep cycles in DoC patients and limited data in some cases, our study might not fully capture the precise characteristics of certain sleep stages. Additionally, we did not observe REM sleep in this patient cohort, which may be due to the inherently low occurrence of REM sleep in these patients or the interruptions caused by routine nighttime care activities. This limitation underscores the challenges of capturing complete sleep architecture in DoC patients under normal clinical conditions. We also did not include a control group of patients under similar conditions. This absence means we lack comparative data on normal sleep patterns and corresponding BIS values in patients without DoC. In future research, we aim to address these limitations by extending the monitoring duration to reflect the sleep–wake patterns of patients more accurately over a longer period. Additionally, we plan to include a control group of normal patients to monitor and compare sleep patterns and BIS values under similar environmental conditions.

## 5 Conclusion

In our study, we observed that patients with DoC exhibit irregular sleep patterns and frequent disruptions, with the BIS effectively differentiating various sleep stages. These findings highlight the need for prolonged monitoring to capture the complex sleep architecture in DoC patients. BIS emerges as a valuable tool for this purpose, offering a less intrusive and practical approach for long-term sleep assessment. However, the lack of significant differences in BIS values across varying consciousness levels suggests that BIS primarily reflects wakefulness rather than cognitive awareness. This research underscores the importance of adapting sleep monitoring techniques to better cater to the specific needs of DoC patients and preliminarily verified that BIS could be used as a suitable tool.

## Data Availability

The raw data supporting the conclusions of this article will be made available by the authors, without undue reservation.

## Footnotes

^1^https://hiplot.com.cn/home/index.html
